# Supplementary material for: A diagnostic primer pair to distinguish between wMel and wAlbB Wolbachia infections
Source: PLoS One. 2021 Sep 23;16(9):e0257781. doi: 10.1371/journal.pone.0257781 (PMC8459989; doi:10.1371/journal.pone.0257781)
Supplement: S1 Table — (DOCX) [file pone.0257781.s001.docx]

# S1 Table. Real-time PCR reagents and volume in 384-well plates with white wells.

| Reagent | Volumes (μL) |
| --- | --- |
| MgCl_2_(50mM) | 0.4 |
| ThermoPol reaction buffer (10X) | 1 |
| High Resolution Melting Master | 0.25 |
| dNTPs(25mM) | 0.064 |
| IMMOLASE^TM^ DNA polymersase (5U/μL) | 0.01 |
| F+R primer combined (50 μM) | 0.08 |
| ddH_2_O | 6.196 |
| DNA template | 2 |
| Total | 10 |
