# Supplementary material for: A diagnostic primer pair to distinguish between wMel and wAlbB Wolbachia infections
Source: PLoS One. 2021 Sep 23;16(9):e0257781. doi: 10.1371/journal.pone.0257781 (PMC8459989; doi:10.1371/journal.pone.0257781)
Supplement: S2 Table — (DOCX) [file pone.0257781.s002.docx]

**S2 Table. Primer efficiency when sample DNA was first diluted six times.**

| Colony | Primers | Slope of graph | Ajusted R^2^ | Efficiency | DNA concentration in Chelex® 100 Resin (ng/µL) | Efficiency curve |
| --- | --- | --- | --- | --- | --- | --- |
| uninfected | *mos* | -1.563 | 0.999 | 101.957% | 9.4 | S2 Fig, a |
| uninfected | *aeg* | -1.507 | 0.998 | 107.302% | 9.4 | S2 Fig, b |
| *w*Mel | *w1* | -1.537 | 0.999 | 104.373% | 6.87 | S2 Fig, c |
| *w*Mel | *wM* | -1.574 | 0.999 | 100.968% | 6.87 | S2 Fig, d |
| *w*Mel | *wsp* | -1.680 | 0.998 | 92.334% | 6.87 | S2 Fig, e |
| *w*Mel | *MA* | -1.747 | 0.999 | 87.546% | 6.87 | S2 Fig, f |
| *w*AlbB | *MA* | -1.753 | 0.999 | 87.123% | 11.73 | S2 Fig, g |
| *w*AlbB | *wsp* | -1.800 | 0.999 | 84.085% | 11.73 | S2 Fig, h |
| *w*AlbB | *wA* | -1.697 | 0.993 | 91.030% | 11.73 | S2 Fig, i |

DNA was extracted in 250 µL 5% Chelex® 100 Resin and then diluted six times before making a three-fold dilution series.
