# Supplementary material for: A diagnostic primer pair to distinguish between wMel and wAlbB Wolbachia infections
Source: PLoS One. 2021 Sep 23;16(9):e0257781. doi: 10.1371/journal.pone.0257781 (PMC8459989; doi:10.1371/journal.pone.0257781)
Supplement: S3 Table — (DOCX) [file pone.0257781.s003.docx]

**S3 Table. Primer efficiency when sample DNA was extracted using different methods.**

| Solution | Colony | Primers | Slope of graph | R^2^ | Efficiency | DNA concentration* (ng/µL) |
| --- | --- | --- | --- | --- | --- | --- |
| Purified from Chelex^®^ 100 Resin | Uninfected | *mos* | -1.656 | 0.996 | 94.141% | 1.80 |
| Purified from Chelex^®^ 100 Resin | Uninfected | *aeg* | -1.679 | 0.989 | 92.384% | 1.80 |
| Purified from Chelex^®^ 100 Resin | *w*Mel | *w1* | -1.866 | 0.995 | 80.174% | 1.30 |
| Purified from Chelex^®^ 100 Resin | *w*Mel | *wM* | -1.799 | 0.985 | 84.187% | 1.30 |
| Purified from Chelex^®^ 100 Resin | *w*Mel | *wsp* | -2.105 | 0.994 | 68.524% | 1.30 |
| Purified from Chelex^®^ 100 Resin | *w*Mel | *wMwA* | -1.914 | 0.993 | 77.560% | 1.30 |
| Purified from Chelex^®^ 100 Resin | *w*AlbB | *wMwA* | -2.168 | 0.965 | 66.792% | 1.25 |
| Purified from Chelex^®^ 100 Resin | *w*AlbB | *wsp* | -2.197 | 0.964 | 64.899% | 1.25 |
| Purified from Chelex^®^ 100 Resin | *w*AlbB | *wA* | -2.148 | 0.944 | 66.792% | 1.25 |
| STE | Uninfected | *mos* | -1.342 | 0.994 | 122.533% | 4.17 |
| STE | Uninfected | *aeg* | -1.387 | 0.991 | 134.082% | 4.17 |
| STE | *w*Mel | *w1* | -1.69 | 0.997 | 96.671% | 5.49 |
| STE | *w*Mel | *wM* | -1.651 | 0.997 | 100.029% | 5.49 |
| STE | *w*Mel | *wsp* | -2.024 | 0.997 | 73.594% | 5.49 |
| STE | *w*Mel | *wMwA* | -1.781 | 0.998 | 94.999% | 5.49 |
| STE | *w*AlbB | *wMwA* | -1.905 | 0.977 | 80.659% | 4.52 |
| STE | *w*AlbB | *wsp* | -2.05 | 0.991 | 70.678% | 4.52 |
| STE | *w*AlbB | *wA* | -1.68 | 0.984 | 92.310% | 4.52 |

*Template DNA was purified from 5% Chelex® 100 Resin or extracted in STE buffer and then diluted ten times before making a three-fold dilution series. Concentration was measured before dilution.
