# Supplementary figures and images for: A diagnostic primer pair to distinguish between wMel and wAlbB Wolbachia infections
Source: PLoS One. 2021 Sep 23;16(9):e0257781. doi: 10.1371/journal.pone.0257781 (PMC8459989; doi:10.1371/journal.pone.0257781)

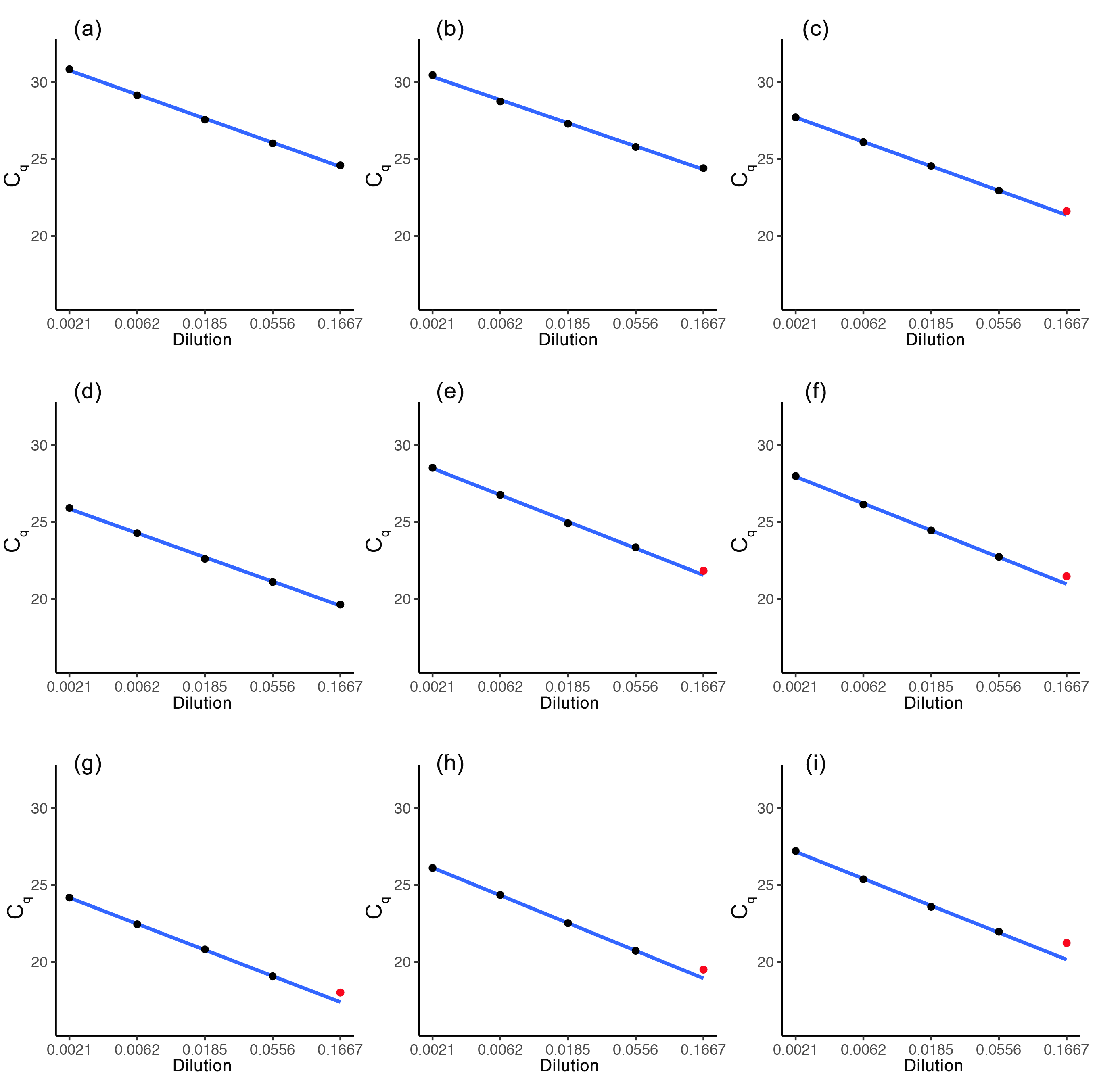

Supplement: S1 Fig — DNA is extracted in 250 μL 5% Chelex® 100 Resin and then diluted six times before making a three-fold dilution series. Outliers are marked with red colour and are excluded from the efficiency curve. The primer names are defined in S3 Table. (PNG) [file pone.0257781.s004.png]

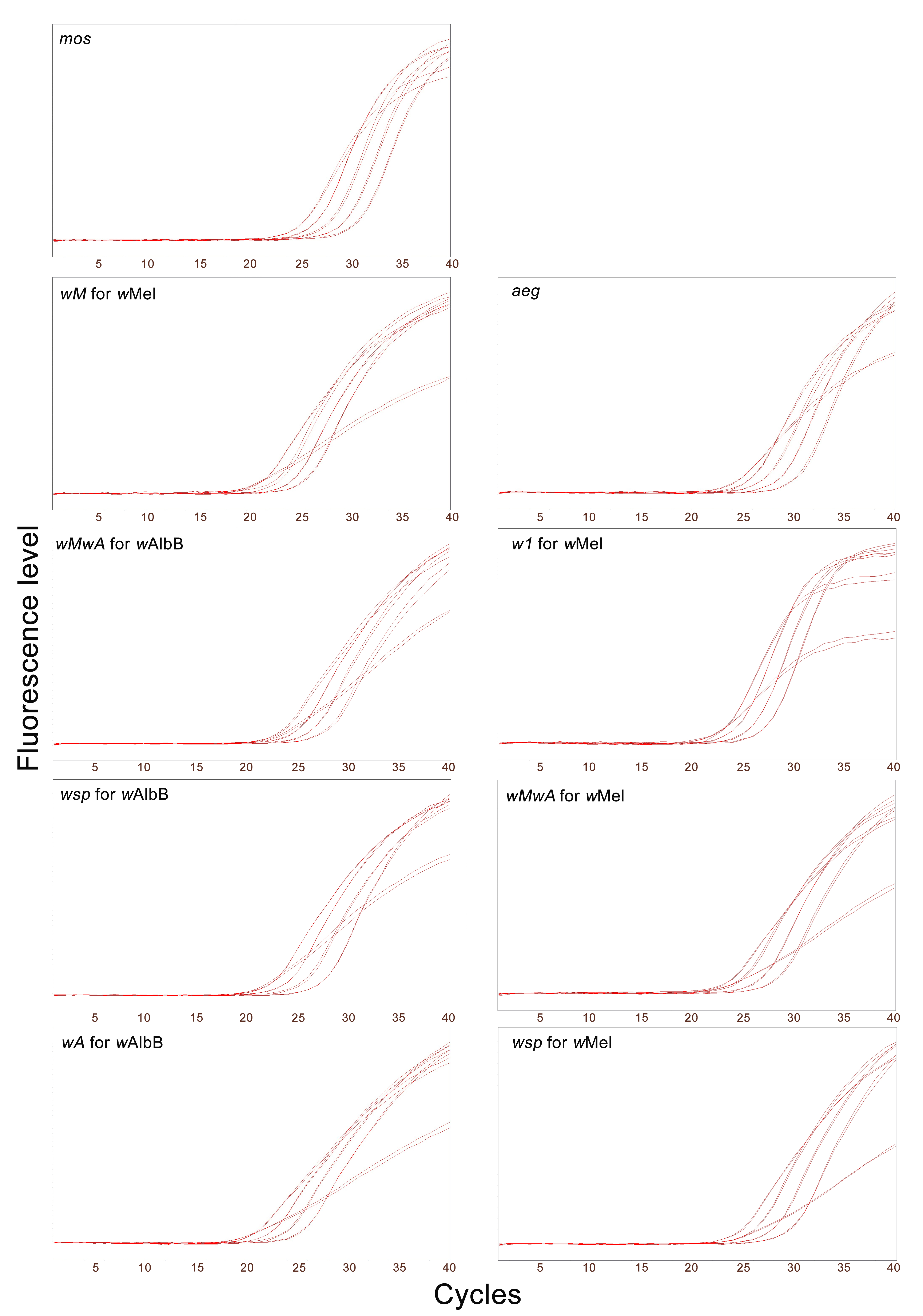

Supplement: S2 Fig — The curves from left to right represent amplification curves of 1/6, 1/18, 1/54, 1/162 and 1/486 DNA dilution from 250 μL 5% Chelex® 100 Resin. The primers are defined in S2 Table. (PNG) [file pone.0257781.s005.png]
