# Supplementary material for: A diagnostic primer pair to distinguish between wMel and wAlbB Wolbachia infections
Source: PLoS One. 2021 Sep 23;16(9):e0257781. doi: 10.1371/journal.pone.0257781 (PMC8459989; doi:10.1371/journal.pone.0257781)
Supplement: Attachment — Submitted filename: Response to Reviewers.docx [file pone.0257781.s006.docx]

1. Is the manuscript technically sound, and do the data support the conclusions?

The manuscript must describe a technically sound piece of scientific research with data that supports the conclusions. Experiments must have been conducted rigorously, with appropriate controls, replication, and sample sizes. The conclusions must be drawn appropriately based on the data presented.

Reviewer #1: Yes

Reviewer #2: Partly

2. Has the statistical analysis been performed appropriately and rigorously?

Reviewer #1: N/A

Reviewer #2: No

3. Have the authors made all data underlying the findings in their manuscript fully available?

The [PLOS Data policy](http://www.plosone.org/static/policies.action#sharing) requires authors to make all data underlying the findings described in their manuscript fully available without restriction, with rare exception (please refer to the Data Availability Statement in the manuscript PDF file). The data should be provided as part of the manuscript or its supporting information, or deposited to a public repository. For example, in addition to summary statistics, the data points behind means, medians and variance measures should be available. If there are restrictions on publicly sharing data—e.g. participant privacy or use of data from a third party—those must be specified.

Reviewer #1: Yes

Reviewer #2: Yes

4. Is the manuscript presented in an intelligible fashion and written in standard English?

PLOS ONE does not copyedit accepted manuscripts, so the language in submitted articles must be clear, correct, and unambiguous. Any typographical or grammatical errors should be corrected at revision, so please note any specific errors here.

Reviewer #1: Yes

Reviewer #2: Yes

5. Review Comments to the Author

Please use the space provided to explain your answers to the questions above. You may also include additional comments for the author, including concerns about dual publication, research ethics, or publication ethics. (Please upload your review as an attachment if it exceeds 20,000 characters)

Reviewer #1: In the manuscript entitled: “A diagnostic primer pair to distinguish between wMel and wAlbB Wolbachia infection”, Lau and colleague’s objective is to improve the real-time PCR diagnostic capabilities of Wolbachia, particularly the wMel and wAlbB strains, currently deployed in field settings around the globe. Based on a gene shared between the two strains (WD_RS06155), and considering the small two-base pair difference between wMel and wAlbB, the authors developed a new primer pair named wMA. Given the data provided, wMA was capable of differentiating between both bacterial strains, while displaying high efficiency and specificity parameters, particularly when compared to commonly primer sets currently employed by the community. I appreciate the concern raised by the authors over the feasibility of this new primer set as a candidate to perform relative measurements of Wolbachia density. Overall, the manuscript is well written, and presented in a clear/easy to understand way. For the most part, experiments are straightforward and well described, with a solid amount of data to support their conclusions. I have listed below, a few suggestions that I believe could improve the quality of the manuscript, prior to acceptance by the editor/Journal.

Comments are displayed in order of appearance:

Line (L) 74: Please provide in this section, more information regarding the gene. If NCBI's designation is correct, the gene is a DNA-directed RNA polymerase subunit beta/beta, with an old locus tag of WD_0024.

**Thank you, we have now added these to the manuscript at lines 80.**

L. 84: Small typo right before “[13]”.

**Deleted**

L. 88: Please specify here if only females were used for the assays, or if it also included males.

**We now added details about the gender of the samples.**

L. 91: Can the authors comment if any other extraction method was used, and if so, how the primers performed on it? For instance, the squash buffer method is commonly employed in certain laboratories as a fast method to detect and quantify Wolbachia.

**We now added comparisons of Chelex extraction to another extraction method (STE buffer extraction) as well as comparing to DNA from a purification of the Chelex®-extracted DNA (methods at lines 124-130). Efficiency results are added in the supplementary information.**

L. 102: Please indicate the final volume used per reaction mix, as well as the final concentration of each primer used. I also encourage the authors to indicate the degree of transparency of the plasticware used, e.g., white or clear, since different plastics exhibit substantial differences in fluorescence reflection and sensitivity.

**We now added the final volume information in the supplementary materials. We clarify that we used 384-well plates with white wells for this assay (SSI Bio, Lodi CA USA, Cat. No. 3430-40).**

L. 107: I encourage the authors to change all designations of “Cp” on the manuscript, to “Cq”. Reasoning for this can be found in the quote below from the widely referenced manuscript for real-time PCR assays: DOI: 10.1373/clinchem.2008.112797

"The nomenclature describing the fractional PCR cycle used for quantification is inconsistent, with threshold cycle (Ct), crossing point(Cp), and take-off point (TOP) currently used in the literature. These terms all refer to the same value from the real-time instrument and were coined by competing manufacturers of real-time instruments for reasons of product differentiation, not scientific accuracy or clarity. We propose the use of quantification cycle (Cq), according to the RDML (Real-Time PCR Data Markup Language) data standard (http:// [www.rdml.org](http://www.rdml.org))"

**Done**

L. 112 – Table 1: Please include on this table, the database accession number for each gene of interest, and the amplicon size for each target gene.

**Done**

L. 122: given the nature of the study in designing a diagnostic primer set, the authors then should include information on how often the assay returns a positive result when a target is present and how often it is negative in the absence of the target. A common requirement of diagnostic assays.

**We have now added this information.**

L. 140: This should not be a big issue, given the results here presented, but I encourage the authors to review their lower-end efficiency value of 85% and the citation used to back up their affirmation. Many distinct publications and guidelines for qPCR experiments encourage a range of 90%-110% as acceptable. One example of such literature can be found at DOI: 10.1016/j.tibtech.2018.12.002

**Thank you, we acknowledge this issue and have now changed the wording and added this reference.**

L. 156 – Fig. 3: Could each curve be represented using a different color, as to facilitate the reader's visualization of the data?

**Done**

Reviewer #2: This manuscript describes design and testing of a qPCR primer annealing to D_RS06155 DNA (directed RNA polymerase subunit beta/beta' gene) of Wolbachia. This primer allows to distinguish between wMel and wAlbB in Aedes aegyptii released to control vector-borne diseases. This primer is also compared to other previously published primers.

The primer's name, wMA, resembles designation of a Wolbachia strain (small "w" and Capital letter following). For clarity, shouldn't it be called something else?

**Thank you, to clarify this we have added an extra letter. Now it is called “*wMwA*”.**

This primers Blast perfectly to wMel (but also to wAu, wYak, wTei, wSan). As wAu has also been transinfected to A. aegyptii, this may limit the utility of this primer for field screening of Aedes. Also, sequences annotated as “Wolbachia endosymbiont of Aedes aegyptii” come up in a blast search quite a lot (eg. CP072672.1), which might be confusing, as these Wolbachia likely do not exist (see Ross et al. 2020). If I was designing the primer, I would avoid the possibility of possibly confusing contaminants. The quick homology searches described above seem important for the usage of the primer, but were not performed by authors and included in the text.

**Thank you, we now added relevant information about the** **test for specificity of this primer pair. However, our primers are targeted for a specific purpose (field released samples), not for *Wolbachia* identification, other *Wolbachia* like *w*Au do not cause CI and are not being used currently for field release so these issues are unlikely to be encountered. Besides, as per Ross et al, we highlight the additional testing required before concluding the presence of *Wolbachia* in a species. We now add relevant information in the manuscript to clarify this.**

Lines 142-146 Shouldn’t samples with contaminants/inhibitors be removed from calibration curves and analysis? How do melting curves for 6x diluted Chelex preps look like?

**Does the reviewer mean amplification curves? The melting curves are consistent and a table with Tm values has been added to the supplementary material. Inhibitors are any factor that prevent the PCR amplification thus it is possible to use different DNA extraction methods or PCR reagents to minimize the impact of inhibitors, but is unlikely to remove them completely. We have now added comparisons of the Chelex extraction with another extraction method. However, contaminants are usually** **caused by human error which obviously** **shouldn’t have occurred in our study as this primer pair had been carefully tested for many times**

Lines 160-168 – is it possible that some primer-targeted sequences have multiple copies within the two genomes? Even if they are all 1-copy sequences this is still expected, as Cp will depend on primer sequence/annealing and amplified fragment sequence.

**All primers used in this study only have one copy based on their genome. Rps genes are single copy in mosquitoes and other insects so this is not an issue (see Lee et al reference cited in the paper). *Wolbachia* are obviously present at multiple copies. The primers are only present as single copies within the *Wolbachia* sequences tested, but it is always possible that there might be amplification of a region of the bacterial genome and we now acknowledge this. This has been shown for the** **octomom region in previous research and we now cite a relevant paper at line 214.**

172-173 – Different number of Wolbachia inside mosquito is to be expected, as this is what the qPCR assay is supposed to test for. The differences within Wolbachia genomes are the issue here. Is WD_RS06155 DNA a multi-copy gene in any of the strains? And, in relation to this also lines 174-175 – can you compare the wMel and wAlbB densities using this primer pairs then?

**In the published genome it is a single copy gene. We now reiterate this but also add a cautionary note. Note similarity in Cp value to *wsp* as in line 222-223**

Finally, the figures presented here have very low resolution.

**We have now checked and increased the resolution.**
